# Rapid Range Shift in an Introduced Tropical Marine Invertebrate

**DOI:** 10.1371/journal.pone.0078008

**Published:** 2013-10-03

**Authors:** Sam Crickenberger, Amy Moran

**Affiliations:** 1 Department of Biological Sciences, Clemson University, Clemson, South Carolina, United States of America; 2 Department of Biology, University of Hawaii, Manoa, Honolulu, Hawaii, United States of America; University of Tasmania, Australia

## Abstract

The barnacle *Megabalanus coccopoma* is native to shorelines from Baja California to Peru and has been introduced to a number of other locations including the Atlantic US SE coast, where it was first recorded in 2006. In 2009, the range of *M. coccopoma* in the SE US extended from Ft. Pierce, FL north to Cape Hatteras, NC with seasonal populations found as far north as Kitty Hawk, NC. During the exceptionally cold winter of 2009/2010, the range of *M. coccopoma* shifted dramatically due to the dieback of all monitored populations north of Florida. We examined body size, distribution, and density of *M. coccopoma* during the summers of 2010, 2011, and 2012 to describe the extent of the range retraction and the rate of range re-expansion. In 2010, recruits were found as far north as Tybee Island, Ga, but no established populations were found north of Florida. In 2011 recruits were found at Rodanthe, NC but established populations were still limited to Florida. By 2012 populations were established in Rodanthe, NC, slightly north of its previously known range limit. Estimated rates of range re-expansion were 255.8 km/yr in 2010 and 794.1 km/yr in 2011. Rates of re-expansion to the north in 2010 and 2011 were faster than have previously been reported for any marine species, and are one of the few rates published for any tropical marine invertebrate.

## Introduction

Poleward range shifts are becoming increasingly common, a phenomenon which often leads to tropical species moving into historically temperate ecosystems [1-3]. Introductions of new species as the result of poleward range shifts can negatively impact ecosystems [4,5] and these range shifts are pervasive in marine environments, consistent with warming trends associated with climate change [6-9]. The frequency of extreme weather events is also predicted to increase with global climate change [10], and extreme warm [11,12] and cold events [13] have been implicated in dramatic decreases in species abundance, but the effects of extreme weather events on range shifts and limits have received less attention than localized consequences of extreme events [14-16].

While tropical species are increasingly becoming established in historically temperate regions [17,18], most studies on tropical invaders have focused on local extinctions rather than range shifts [19,20] and little is known about lower thermal tolerances of tropical species, which likely set northern range limits of these species as the tropical belt broadens [21-24]. The climate variability hypothesis predicts that, due to the relative stability of tropical climates, tropical species will have narrower windows of thermal tolerance than temperate species [25]. Likewise, because of the relative lack of seasonal variation in temperature in the tropics, tropical species are generally thought to have a lower capacity for acclimation than temperate species [26]. Tropical species that have narrow thermal tolerance windows and low acclimatory ability may be particularly vulnerable to extreme cold events, and therefore invasive populations of tropical species may be generally more ephemeral than temperate species when both are moving poleward.

On the Atlantic SE coast of the US, the ranges of a number of species originating in the tropics have been expanding northward [2,27-29]. Over the same time frame, the number of extremely cold days has increased in frequency along the Atlantic coast of the US SE [30]; these cold events may play a role in regulating the dynamics of tropical species that have invaded temperate ecosystems. During the winter of 2009/2010 air temperatures were the coldest they have been in more than 30 years throughout the US SE [31] and seawater temperatures were colder than those in the past 20 years (South Carolina Department of Natural Resources-Marine Resources Division, unpublished data). The exceptionally cold temperatures during the winter of 2009/2010 were associated with the lowest Arctic Oscillation index (AO) and the 5^th^ highest El Nino Southern Oscillation (ENSO) index recorded [31], and were implicated in localized die-offs of three different tropical marine invertebrates in the US SE including the crab *Petrolisthes armatus* [22] and the mussels *Perna viridis* [20] and *Mytella charruana* [33]; *P. armatus* and *P. viridis* have experienced high mortality during previous cold winters [19,20]. Here we report the range retraction of the barnacle *Megabalanus coccopoma* following the cold winter of 2009/2010 and the species’ very rapid subsequent range re-expansion.

## Methods

### Study species

*Megabalanus coccopoma* is a highly gregarious acorn barnacle native to coasts extending from the southern tip of Baja California, Mexico to Peru [34] that commonly attaches to recently disturbed surfaces in the lower intertidal or subtidal [35]. *M. coccopoma* has been a successful invader of nearshore marine systems in many locations worldwide. The earliest report of this species outside of its native range came from the western Indian Ocean in 1875 [36]. In the 1970s *M. coccopoma* was reported from the western Atlantic in Brazil [34,37,38] and it was first documented in the United States in 2001 when several individuals were found near Jefferson, LA; these individuals did not survive the winter [39]. In 2006, *M. coccopoma* was found for the first time on the SE coast outside of the Gulf of Mexico when it appeared in Florida, Georgia, South Carolina, and North Carolina (http://nas.er.usgs.gov/). Populations of *M. coccopoma* are now well established in the SE United States since 2007 ( [29,40]; S. Crickenberger pers. obs.), along with Brazil since the 1970s [41-46], the Atlantic coast of Mexico since at least 2005 [47], southern Japan since 2005, the east coast of Australia since at least 2006 [48], and western Africa since at least 2010 [49]. Records from the North Sea off the coasts of Belgium and the Netherlands suggest the presence of sporadic and seasonal populations there [50-53] and seasonal populations of *M. coccopoma* were reported in Southern California during an El Niño year [54].

### Sites and survey methods

Surveys of body size and density of *M. coccopoma* were conducted from 16 July to 30 July 2010, 25 July to 10 August 2011, and 25 July to 11 August 2012 to determine the distribution and abundance of juvenile and adult *M. coccopoma* along the SE US coast. In this region, recruitment of *M. coccopoma* peaks in May to June and then tails off through August ( [40]; S. Crickenberger pers. obs.), therefore our sampling schedule allowed us to quantify both presence and abundance of new recruits and to clearly separate young-of-the-year from barnacles from previous recruitment seasons based on size (see below). Density (barnacles/cm^2^) and basal rostro-carinal diameter (mm) (as an estimate of size) of *M. coccopoma* were recorded at 14 locations in 2010 and 19 locations in 2011 and 2012 between the northernmost and southernmost extents of the range of *M. coccopoma* in the Atlantic SE US (Table 1). Basal rostro-carinal diameters of all barnacles collected from all the quadrat(s) at each site were measured with calipers. One to four sites within each location were sampled (Table 1). At sites with large areas of continuous substrate (i.e. floating docks, jetties, buoys, beach groins), three to six quadrats of 100 cm^2^ were sampled every half to one meter along a 10 m transect laid in the zone of maximum density. At sites where substrate was discontinuous (i.e. pier pilings), three to six 100 cm^2^ quadrats were sampled in the zone of maximum density. When no *M. coccopoma* were sampled in any quadrats or when only a single structure was present (i.e. channel markers), all of the *M. coccopoma* on the structure(s) were collected and the area of the structure(s) was quantified. If no *M. coccopoma* were found during the initial search the site was subsequently searched for the lesser of 30 minutes or the time required to survey the entire structure. If no specimens were found in this second search, *M. coccopoma* was considered absent from that site. Low availability of hard substrate limited the number of potential quadrats at some sites. Sapelo Island National Estuarine Research Reserve (NERR) granted permission for collection in Sapelo Island NERR and Al Segars granted permission for sampling in ACE Basin NERR. Permission was granted for privately owned floating docks on the day of collection. Other sites did not require permission for collection because *M. coccopoma* is not an endangered or protected species and/or the sites were not privately owned or protected.

**Table 1:** Locations and sites sampled for *Megabalanus coccopoma* in 2010, 2011, and 2012.

**Location**	**Abbreviation**	**Site °N**	**Site °W**	**Years Surveyed**
Ft. Pierce, FL	FTP	27°28.273'	080°17.234'	2010, 2011, 2012
		27°27.658'	080°19.008'	2010, 2011, 2012
		27°27.654'	080°19.013'	2011, 2012
Ponce Inlet, FL	PON	29°04.698'	080°54.996'	2010, 2011
		29°04.884’	080°56.125'	2010, 2011, 2012
Daytona Beach, FL	DBP	29°13.653'	081°00.319’	2011, 2012
GTMNERR, FL	GTM	29°42.380'	081°13.793'	2010, 2011
		29°41.975'	081°13.936'	2010
		29°43.398'	081°14.736'	2011, 2012
		29°42.943'	081°14.354’	2010, 2011, 2012
Flagler Beach, FL	FLG	29°28.812'	081°07.527'	2011, 2012
Jacksonville Beach, FL	JAK	30°23.784'	081°25.799'	2011
		30°17.614'	081°23.263'	2011, 2012
Fernandina Beach, FL	FER	30°30.636'	081°27.655’	2011, 2012
		30°37.240'	081°26.272’	2011, 2012
Saint Simons Island, GA	SSI	31°08.022'	081°23.734'	2010, 2011, 2012
Sapelo Island NERR, GA	SAP	31°25.083'	081°17.779'	2011, 2012
		31°32.422'	081°14.544'	2011, 2012
		31°32.476'	081°10.697'	2011, 2012
Tybee Island, GA	TYB	31°59.494'	080°50.698'	2010, 2011, 2012
Hunting Island, SC	HBG	32°21.563'	080°25.527'	2010, 2011, 2012
ACE Basin NERR, SC	ACE	32°29.610'	080°21.005'	2010, 2011, 2012
		32°28.063'	080°20.066'	2011, 2012
Folly Beach, SC	FBP	32°39.219'	079°56.333'	2010, 2011, 2012
Murrells Inlet, SC	MUR	33°31.537'	079°01.811'	2010
		33°34.560'	078°59.845'	2011, 2012
Wrightsville Beach, NC	WBP	34°12.837'	077°47.270'	2010, 2011, 2012
		34°11.589'	077°48.250'	2011, 2012
Frisco, NC	FRP	35°13.512'	075°38.161’	2010, 2011, 2012
Avon, NC	AVP	35°20.839'	075°30.026'	2010, 2011, 2012
Rodanthe, NC	ROP	35°35.100'	075°27.648'	2010, 2011, 2012
Kill Devil Hills, NC	KDH	36°02.663'	075°40.454'	2010, 2011, 2012

### Population distribution

Populations of *M. coccopoma* at each location were classified as either established, present, or absent based on sizes of the barnacles (if present) at that location. Populations were considered established if any barnacles at that location were larger than the largest size the barnacles could reach since the beginning of the settlement season based on growth observations from 2012 (see below). To measure growth rates of individual barnacles and to estimate the size barnacles could reach in one settlement season, six quadrats (10 x 10 cm) were established at Flagler Beach Pier (29°28.812' N, 081°07.527' W), within the zone of maximum density of *M. coccopoma*. Three of the plots were entirely cleared of barnacles and three were partially cleared, leaving a clump of existing individuals in the center of the quadrats to allow tracking of barnacle growth and survivorship of new recruits. Plots were marked at each corner with carriage bolts screwed into the pier pilings. Quadrats were photographed monthly from 3 April 2012 to 28 July 2012 using a digital camera (Nikon Coolpix AW100) and a PVC frame placed over the carriage bolts to ensure consistent camera distance for size measurements. Rostro-carinal basal diameter was measured from digital photographs with ImageJ [55] on all barnacles in each of 30 total quadrats. All newly settled barnacles (within the last month) were individually followed for growth. Across all quadrats, 16 barnacles that settled early in the spawning season survived to the last sampling period. Using the mean and standard deviation of this sample, we used JMP 10.0 (Version 10, SAS Institute Inc., Cary, NC, USA) to calculate the 99% upper one-sided tolerance interval with 99% confidence; this allowed us to establish a conservative lower size limit for second-year barnacles such that we could say, with 99% confidence, that there was a 99% chance that a barnacle above that size was too large to be from the current recruitment season. Populations were categorized as established if the size of any barnacles present in that population was above the upper tolerance interval. Populations were categorized as present if all barnacles at that site were below the upper tolerance interval. Determination of absence is described above. Where possible we sampled multiple sites within locations and size data were pooled among sites to determine whether each location was categorized as established or present; data were pooled because we were interested in broad, rather than local, patterns of colonization and extinction. No quantitative survey data were available for 2009, so distributional data for this year was gathered from sources listed in Table S1.

### Population density

The site with the greatest density at each location was used to represent that location, to avoid sampling artificially low densities from sites where all *M. coccopoma* were removed in the previous year. In locations where three years of data were collected, density data were non-normal and variances were heterogeneous with the exceptions of PON and FTP. Therefore, maximum density within each location was compared among years using Wilcoxon/Kruskal-Wallis tests followed by Wilcoxon paired comparisons when significant differences among years were found. In locations where data were only collected for 2011 and 2012, data were normally distributed and variances were homogenous, so paired t-tests were used to compare densities between years. Maximum density data from FER were transformed by adding one to all values followed by log transformation to achieve normality. Low numbers of quadrats (due to the limited availability of hard substrate at some sites) limited our degrees of freedom within sites and prohibited examining all locations in a single analysis. All statistical analyses were performed in JMP (Version 10, SAS Institute Inc., Cary, NC, USA).

### Range shifts

Range shifts between years were calculated as great circle distances using the Vincenty formula, which calculates the distance between two points on an ellipsoidal model of the earth [56]. The range retraction that occurred during the winter of 2009/2010 was estimated by calculating the distance between the most northern population known from 2009, at Avon, NC (35°20.839' N, 075°30.026' W), and the most northern established population found during the summer of 2010 at St. Augustine, FL (29°42.943' N, 081°14.354' W). The range re-expansion that occurred over the summer of 2010 was calculated as the distance from St. Augustine, FL to the most northern location where *M. coccopoma* were present (at Tybee Island, GA (31°59.494’ N, 080°50.698’ W)) in 2010. For the summer of 2011, range re-expansion was calculated as the distance from the northernmost established population found in the summer of 2011 at Fernandina Beach, FL (30°30.636' N, 081°27.655’ W) to the northernmost present population found in the summer of 2011 at Rodanthe, NC (35°35.100’ N, 075°27.648’ W).

### Environmental temperatures

Temperature data from January and February of 2009, 2010, 2011 and 2012 were obtained from the National Data Buoy Center (http://www.ndbc.noaa.gov/) and the National Estuarine Research Reserve System Central Data Management Office (http://cdmo.baruch.sc.edu/get/export.cfm). Temperature data from each station were compiled into daily means and used to calculate average winter (January and February) temperatures for each year at each station. Maximum and minimum temperatures were the maximum and minimum daily means that occurred at each station in each year.

## Results

### Upper tolerance limit for size of year-old barnacles

**Figure 1:**
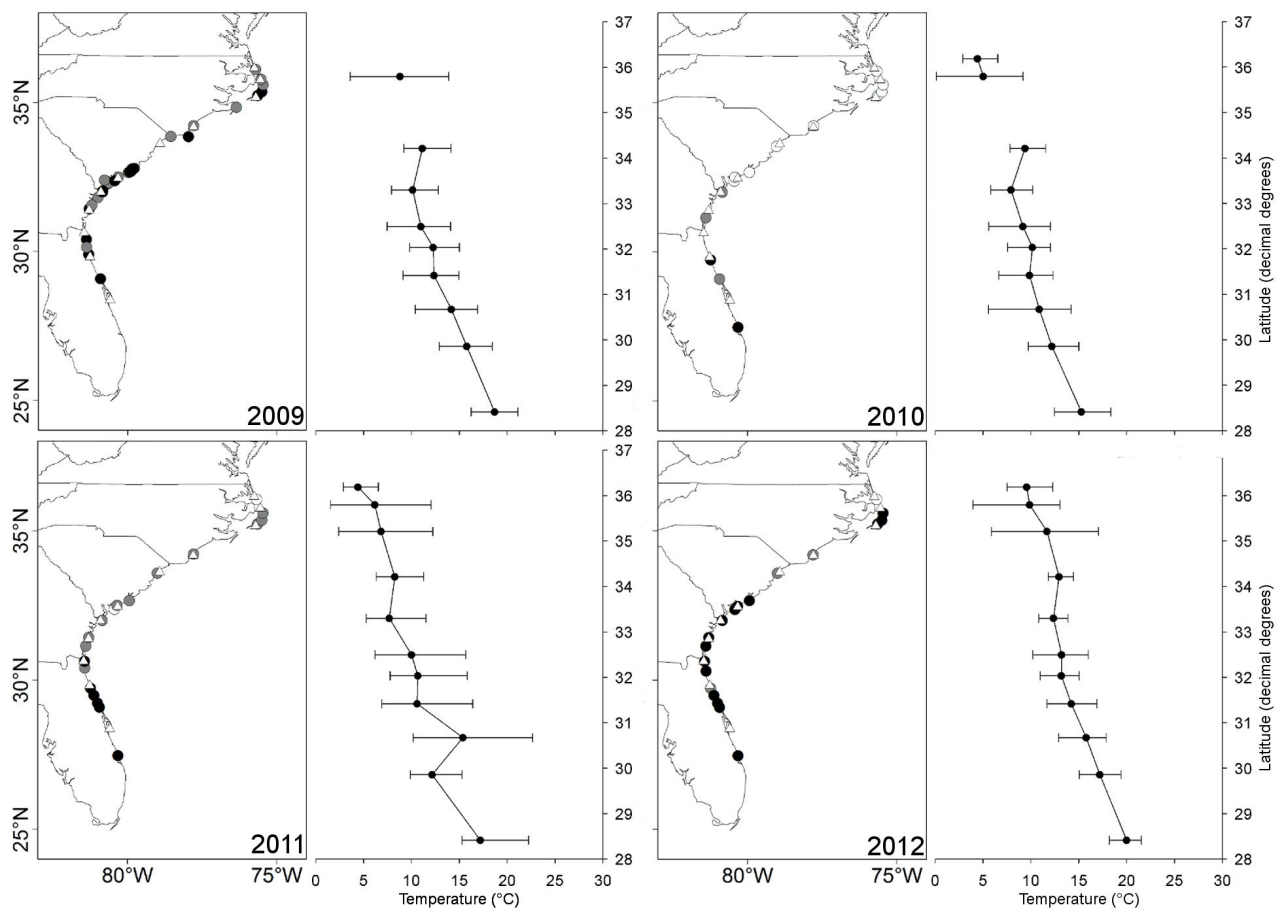
Distribution of *Megabalanus coccopoma* in the US SE. The distribution of *M. coccopoma* in 2009, 2010, 2011, and 2012 showing populations that, in each year, were established (black circles), present but not established (gray circles), or absent (light gray circles). Locations for temperature stations are marked with white triangles. Temperatures are the daily mean January and February temperatures for each station with bars representing the maximum and minimum recorded daily temperatures. Temperature records were unavailable for two locations in 2009 and one location in 2010.

The 16 barnacles that settled early in the spawning season (May) and survived to the last sampling period (28 July 2012) had a mean size of 13.6 mm (± 0.5 mm SD) basal rostro-carinal diameter. The 99% upper one-sided tolerance interval with 99% confidence was 32.8 mm, which was 12.6 mm greater than the size of the largest barnacle in the group of 16. All barnacles greater than the upper one-sided tolerance interval of 32.8 mm were considered to have settled in the previous recruitment season and locations where these larger barnacles were found were termed as established populations (Figure 1; Table S2).

### Distribution

After the cold winter of 2009/2010, *M. coccopoma* died off at all study sites north of Florida. During the summers of 2010 and 2011 the range of *M. coccopoma* rapidly expanded poleward. In summer 2010, *M. coccopoma* was present but not established from Tybee Island south to St. Augustine, FL (29°42.943' N, 081°14.354' W), but was absent from sites north of Tybee Island, GA (31°59.494’ N, 080°50.698’ W). Established populations were found south of St. Augustine, FL, with the exception Ponce Inlet (29°04.884' N, 080°56.125' W). In 2011, new recruits were found as far north as Rodanthe, NC (35°35.100’ N, 075°27.648’ W) with established populations still limited to Florida. In 2012, established populations were found as far north as Rodanthe, NC, slightly further north than their previously reported northernmost limit at Avon, NC (35°20.848’ N, 75°30.110’ W) (Table S1, S2). Established populations were not continuously distributed throughout their full range. At two sites that were south of the northernmost established population, only new recruits were found at Murrells Inlet, SC (33°34.560’ N, 078°59.845’ W) and Wrightsville Beach, NC (34°12.837’ N, 077°47.270’ W) (Figure 1).

### Density

**Figure 2:**
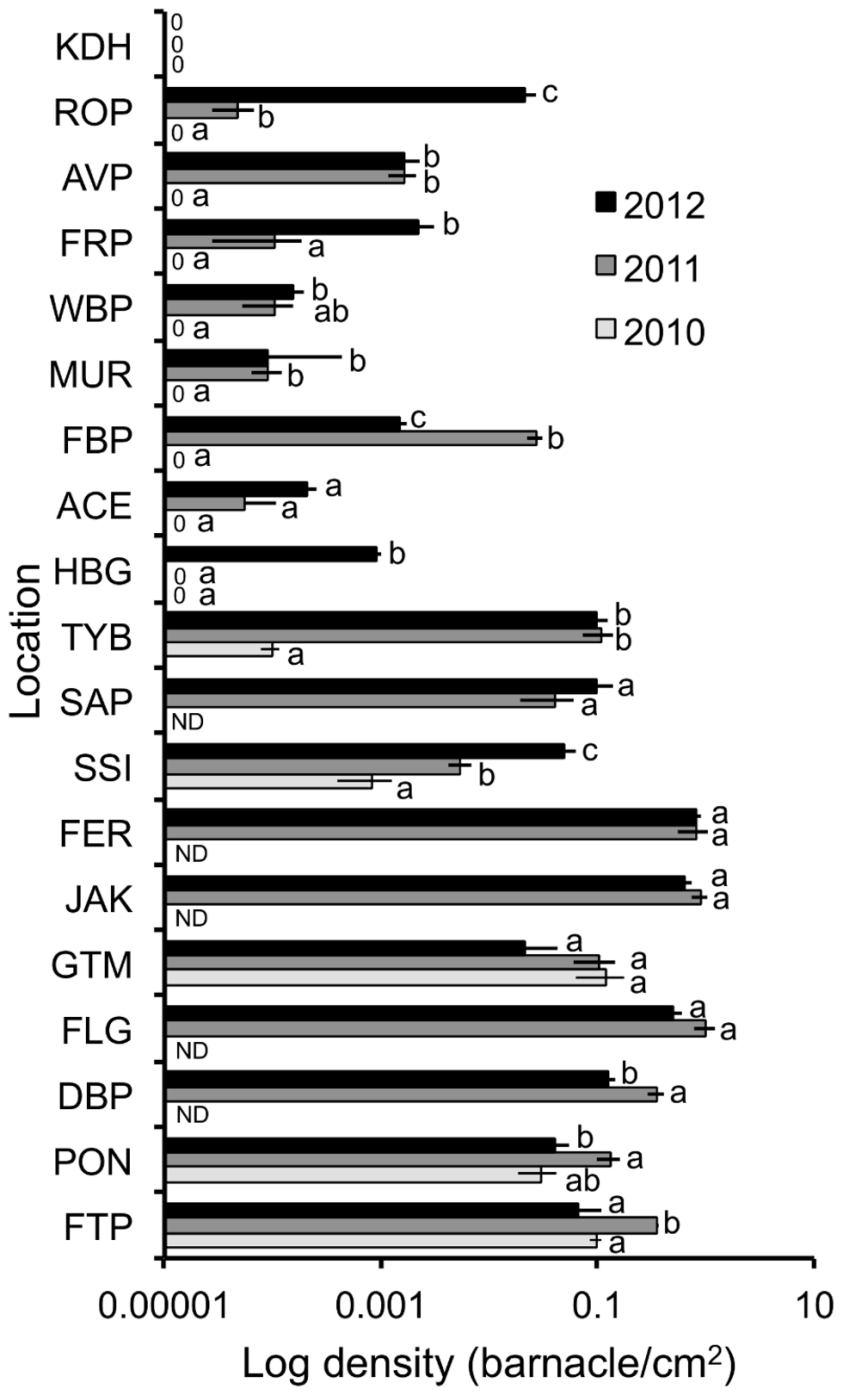
Log of maximum density of *Megabalanus coccopoma* at each location in 2010, 2011, and 2012. Location abbreviations represent locations in increasing latitude as listed in Table 1. ND represents locations that were not sampled in 2010 and ‘0’ represents locations where *Megabalanus coccopoma* was absent. Bars within a location are not significantly different if they share the same letter.

Density increased at most locations north of Florida from one summer to the next in 2010, 2011 and 2012. In 2010, low densities of *M. coccopoma* were found at St. Simons Island, GA (31°08.022'N, 081°23.734'W) and Tybee Island, GA (31°59.494'N, 080°50.698'W). Compared to 2010, densities significantly increased at all locations north of Florida in 2011 except for Hunting Island, SC (32°21.563'N, 080°25.527'W), ACE Basin NERR, SC (32°29.610'N, 080°21.005'W), Wrightsville Beach, NC (34°12.837'N, 077°47.270'W) and Frisco, NC (35°13.512'N, 075°38.161'N) where densities remained unchanged. Although *M. coccopoma* was present at ACE Basin NERR, Wrightsville Beach, NC and Frisco, NC in 2011, densities were not significantly different from zero. Densities were significantly higher at four locations in Georgia, South Carolina, and North Carolina in 2012 compared to 2011. Density significantly increased at St. Simons Island, GA, Hunting Island, SC, Frisco, NC and Rodanthe, NC (35°35.100'N, 075°27.648'N). Densities at other locations remained the same, with the exception of Folly Beach, SC (32°39.219'N, 079°56.333'N), where density decreased (Figure 2; Table 2).

**Table 2:** Effect of year on maximum *Megabalanus coccopoma* density at each location.

**Location**	**Chi-square/ t-Ratio**	**DF**	**Prob>Chi-square/ Prob> |t|**
FTP	7.5114	2	0.0234
DBP*	-3.41419	5.846323	0.0148
PON	7.4583	2	0.024
GTM	4.7849	2	0.0914
FLG*	-2.11132	7.042936	0.0724
JAK*	-1.32702	8.797383	0.2179
FER*	0.263084	9.45832	0.7981
NEP	8.857	2	0.029
SAP*	1.333989	3.130966	0.2709
TYB	8.5118	2	0.0142
HBG	10.4554	2	0.0054
ACE	4.633	2	0.0986
FBP	13.4731	2	0.0012
MUR	6.8	2	0.0334
WBP	7.3194	2	0.0257
FRP	10.2785	2	0.0059
AVP	8.2904	2	0.0158
ROP	10.8833	2	0.0043

* Denotes locations where paired comparisons were made between two years (2011 and 2012). At all other locations all three years were compared.

### Rates of range shifts

In 2009, the range of *M. coccopoma* extended north to Avon, NC (35°20.839' N, 075°30.026' W) with some seasonal populations as far north as Kitty Hawk, NC (36°6.070’ N, 075°42.698’ W). The exceptionally cold winter of 2009/2010 caused local extinctions of all *M. coccopoma* at study sites north of Florida (Figure 1), and in the following two summers (2010 and 2011) the species’ range rapidly re-expanded northward. The estimated range retraction during the winter of 2009/2010 was 825.2 km. The estimated range re-expansion rate was 255.8 km/yr in 2010 and 794.1 km/yr in 2011. In 2012 range limits were unchanged from 2011.

## Discussion

After the dieback during the winter of 2009/2010, range re-expansion by *M. coccopoma* was rapid in both 2010 and 2011; the 2011 rate was more than three times higher than the most rapid rate documented for any marine invertebrate, 235 km/yr for the mussel *Perna perna* [57]. Coastal currents, in concert with warmer temperatures during the winter of 2011/2012, likely played a major role in the rapid range re-expansion of *M. coccopoma* (Fig. 1). Currents important for larval transport along the US SE coast are primarily wind driven and vary seasonally [58,59]. Coastal currents are rotating towards and then flowing poleward along the Atlantic SE coast [60-62] during the peak recruitment period of *M. coccopoma* from May to July, and drifter data suggest transport from central Florida to the Outer Banks of North Carolina is possible in as few as 15 days [40,63]. These wind-driven currents could have facilitated the rapid range re-expansion of *M. coccopoma* and may also explain why the first reports of this species in the SE US in 2006 were spread over a wide geographic area including sites in Florida, Georgia, South Carolina, and North Carolina (http://nas.er.usgs.gov).

While current regimes are likely to have played an important role, the rapid range re-expansion of *M. coccopoma* may also have been facilitated by the species’ high fecundity, rapid maturation, and aggregative settlement. *M. coccopoma* typically release 30,0000 nauplii per spawning event, while other acorn barnacles produce around 6,000 (Crickenberger, unpublished data [35]; and sources within). Reproduction begins at a young age; gametic tissue can be found in barnacles with rostro-carinal basal diameters as small as 8.7 mm (~1 month old), with nauplii hatching from barnacles as small as 19.6 mm in rostro-carinal basal diameter (~3 months old) (Crickenberger, unpublished data). This suggests barnacles that recruit in the spring could reach reproductive maturity within a single season, potentially contributing larvae to the second, smaller peak in recruitment in October and November (pers. obs; [40]). Likewise, where *M. coccopoma* is abundant, barnacles typically grow on top one another in large aggregated clumps. Dense clumps can provide access to mates at the range edge and alleviate Allee effects that could otherwise slow rates of range expansion [64]. Allee effects are likely to be particularly strong in sessile animals with internal fertilization such as *M. coccopoma* [65,66].

Anthropogenic factors could also have played a role in the rapid range re-expansion. *M. coccopoma* was introduced to Brazil in the 1970s, probably arriving on ship hulls. Ships from Brazil with *M. coccopoma* attached to their hulls likely traveled to the US Gulf and the Atlantic coast of Florida, allowing the barnacles to release their larvae which then developed and settled in these regions during the initial introduction of *M. coccopoma* to the US SE [39,45]. However, the increases in density along the coast between the summers of 2010, 2011 and 2012 (Figure 2; Table 2, S2) are consistent with natural dispersal via seasonal patterns of poleward currents during spawning rather than with repeated introductions on ship hulls.

Marine species are projected to continue moving northward along the Atlantic SE coast of the US with climate change [67]. However, the number of extremely cold days has increased in frequency along the Atlantic coast of the US SE [30] and extreme cold events are predicted to increase in frequency [32]. These cold events may play a role in regulating the dynamics of tropical species that have made their way into temperate ecosystems. In contrast to tropical species, which undergo localized extinctions in response to extreme cold weather events [19,68,69], temperate species tend to respond to colder than average winters through recruitment failure and reductions in adult density [13,70]. Only rarely have localized extinctions due to extreme cold events occurred in temperate species [14]. Limited selection for cold tolerance mechanisms in the tropics, narrow thermal tolerance windows, and low capacity for acclimation may make tropical species particularly vulnerable to cold mortality [26,71,72]. Based on temperatures at which localized die-offs occurred during the winter of 2009/2010, *M. coccopoma* lives near its lower thermal limits within the temperate portion of its invaded range in the US SE (Figure 1). Similarly cold winters have occurred multiple times in the past [19,20,73-75] and are likely to occur in the future [32]. Although *M. coccopoma* was able to rapidly re-colonize throughout the extent of its range retraction, rates of range expansion of other species with more limited dispersal and colonization capabilities and/or less serendipitous timing of reproduction will likely be lower. Future projections of species range shifts due to climate change should incorporate the likely effects of extreme cold events on species moving out of the tropics into temperate regions.

## Supporting Information

Table S1 **List of locations and sources for *Megabalanus coccopoma* prior to the range retraction during the winter of 2009/2010.** Populations were considered established if listed as such on the USGS NAS database or if large, dense aggregations were known to be present prior to the cold snap from various unpublished sources. Otherwise *Megabalanus coccopoma* was considered present at a given location.
(DOCX)
(DOCX)

Table S2 **Size of *Megabalanus coccopoma* at locations surveyed in 2010, 2011, and 2012.**
(DOCX)
(DOCX)
