# Supplementary material for: Rapid Range Shift in an Introduced Tropical Marine Invertebrate
Source: PLoS One. 2013 Oct 3;8(10):e78008. doi: 10.1371/journal.pone.0078008 (PMC3789662; doi:10.1371/journal.pone.0078008)
Supplement: Table S1 — List of locations and sources for Megabalanus coccopoma prior to the range retraction during the winter of 2009/2010. Populations were considered established if listed as such on the USGS NAS database or if large, dense aggregations were known to be present prior to the cold snap from various unpublished sources. Otherwise Megabalanus coccopoma was considered present at a given location. (DOCX) (DOCX) [file pone.0078008.s001.docx]

Table S1: List of locations and sources for *Megabalanus coccopoma* prior to the range retraction during the winter of 2009/2010. Populations were considered established if listed as such on the USGS NAS database or if large, dense aggregations were known to be present prior to the cold snap from various unpublished sources. Otherwise *Megabalanus coccopoma* was considered present at a given location.

| **Location** | **˚N** | **˚W** | **Status** | **Date** | **Source** |
| --- | --- | --- | --- | --- | --- |
| Ponce Inlet, FL | 29.0724846 | -80.918664 | established | 2006 | USGS NAS |
| St. Augustine, FL | 29.8917 | -81.31 | established | 2006 | USGS NAS |
| Palm Valey, FL | 30.1327 | -81.3851 | present | 2007 | USGS NAS |
| Jacksonville, FL | 30.397383 | -81.390409 | established | 2007 | USGS NAS |
| Sapelo Island, GA | 31.41805 | -81.29631667 | established | 2009 | D. Hurely pers. comm. |
| Sapelo Island, GA | 31.54832 | -81.21068 | present | 2009 | J. Shalack pers. comm. |
| Chatham, GA | 31.8042 | -81.0042 | present | 2006 | USGS NAS |
| Tybee Island GA | 31.99146667 | -80.84495 | established | 2009 | J. Richardson pers. comm. |
| Tybee Island, GA | 32.02419167 | -80.84133611 | established | 2009 | J. Richardson pers. comm. |
| Beaufort, SC | 32.261868 | -80.678163 | established | 2007 | USGS NAS |
| Beaufort, SC | 32.29083333 | -80.64583333 | established | 2009 | D. Knott pers. comm. |
| Beaufort, SC | 32.30694444 | -80.64583333 | established | 2009 | D. Knott pers. comm. |
| Hunting Island, SC | 32.3624 | -80.44073333 | established | 2009 | pers. obs. |
| Beaufort, SC | 32.38429167 | -80.78694444 | established | 2009 | D. Knott pers. comm. |
| Edisto Beach, SC | 32.4872 | -80.3197 | present | 2007 | USGS NAS |
| Folly Beach, SC | 32.639625 | -79.979535 | present | 2006 | USGS NAS |
| Folly Beach, SC | 32.65365 | -79.93888333 | established | 2009 | pers. obs. |
| Folly Beach, SC | 32.6857 | -79.8867 | established | 2006 | USGS NAS |
| Sullivans Island, SC | 32.7583 | -79.8597 | established | 2006 | USGS NAS |
| Isle of Palms, SC | 32.78428333 | -79.78513333 | established | 2009 | pers. obs |
| Bald Head Island, NC | 33.840336 | -77.964993 | established | 2006 | USGS NAS |
| Waties Island, SC | 33.8483 | -78.5542 | present | 2007 | USGS NAS |
| Wrightsville Beach, NC | 34.21166 | -77.790241 | present | 2006 | USGS NAS |
| Carteret, NC | 34.82973 | -76.35473 | present | 2007 | USGS NAS |
| Frisco, NC | 35.2254167 | -75.6361389 | established | 2007 | S. Jones pers. comm. |
| Avon, NC | 35.347472 | -75.50183 | established | 2007 | S. Jones pers. comm. |
| Rodanthe, NC | 35.585 | -75.4608 | present | 2007 | S. Jones pers. comm. |
| Oregon Inlet, NC | 35.76683 | -75.527583 | present | 2007 | S. Jones pers. comm. |
| Kitty Hawk, NC | 36.101167 | -75.7116389 | present | 2007 | S. Jones pers. comm. |
