# Supplementary material for: Rapid Range Shift in an Introduced Tropical Marine Invertebrate
Source: PLoS One. 2013 Oct 3;8(10):e78008. doi: 10.1371/journal.pone.0078008 (PMC3789662; doi:10.1371/journal.pone.0078008)
Supplement: Table S2 — Size of Megabalanus coccopoma at locations surveyed in 2010, 2011, and 2012. (DOCX) (DOCX) [file pone.0078008.s002.docx]

Table S2: Size of *Megabalanus coccopoma* at locations surveyed in 2010, 2011, and 2012.

| Location | Year | n | Mean (mm) | SE | Range (mm) | # of individuals > 32.8 mm |
| --- | --- | --- | --- | --- | --- | --- |
| Ft. Pierce, FL | 2010 | 82 | 17.1 | 1.0 | 45.5 - 4.7 | 5 |
|  | 2011 | 245 | 19.2 | 0.6 | 45.8 – 1.5 | 18 |
|  | 2012 | 70 | 21.4 | 1.1 | 47.3 – 2.6 | 7 |
| Ponce Inlet, FL | 2010 | 59 | 14.2 | 1.1 | 30.4 – 3.5 | 0 |
|  | 2011 | 78 | 18.6 | 0.9 | 44.6 – 5.5 | 5 |
|  | 2012 | 24 | 22.3 | 2.0 | 38.1 – 7.8 | 4 |
| Daytona Beach, FL | 2010 | - | - | - | - | - |
|  | 2011 | 211 | 9.2 | 0.2 | 38.8 – 2.5 | 1 |
|  | 2012 | 77 | 19.7 | 1.2 | 41.7 – 1.8 | 8 |
| Flagler Beach, FL | 2010 | - | - | - | - | - |
|  | 2011 | 694 | 12.2 | 0.3 | 55.9 - 2.3 | 12 |
|  | 2012 | 294 | 17.7 | 0.6 | 51.2 – 4.8 | 34 |
| GTMNERR, FL | 2010 | 166 | 14.4 | 0.8 | 45.7 – 2.1 | 13 |
|  | 2011 | 78 | 11.4 | 0.6 | 51.3 – 2.4 | 2 |
|  | 2012 | 13 | 11.9 | 1.4 | 21.2 – 5 | 0 |
| Jacksonville Beach, FL | 2010 | - | - | - | - | - |
|  | 2011 | 535 | 6.6 | 0.1 | 32.6 – 0.8 | 0 |
|  | 2012 | 385 | 13.5 | 0.3 | 36.1 - 2 | 2 |
| Fernandina Beach, FL | 2010 | - | - | - | - | - |
|  | 2011 | 458 | 8.1 | 0.2 | 39.2 - 1 | 2 |
|  | 2012 | 469 | 13.8 | 0.4 | 70.7 - 1 | 16 |
| Saint Simons Island, GA | 2010 | 23 | 8.5 | 0.7 | 15.3 - 2 | 0 |
|  | 2011 | 144 | 13.0 | 0.3 | 20.4 – 7.1 | 0 |
|  | 2012 | 637 | 12.9 | 0.2 | 46.4 – 1.6 | 6 |
| Sapelo Island NERR, GA | 2010 | - | - | - | - | - |
|  | 2011 | 28 | 16.7 | 1.2 | 27.2 – 4.2 | 0 |
|  | 2012 | 462 | 21.0 | 0.3 | 66.1 – 5.1 | 12 |
| Tybee Island, GA | 2010 | 6 | 14.1 | 1.5 | 20.9 – 9.8 | 0 |
|  | 2011 | 124 | 16.4 | 0.5 | 28.3 - 3.3 | 0 |
|  | 2012 | 56 | 26.4 | 1.0 | 55.0 – 10.0 | 9 |
| Hunting Island, SC | 2010 | 0 | - | - | - | - |
|  | 2011 | 0 | - | - | - | - |
|  | 2012 | 72 | 19.8 | 0.5 | 38.0 – 11.8 | 2 |
| ACE Basin NERR, SC | 2010 | 0 | - | - | - | - |
|  | 2011 | 1 | - | - | 16.2 | 0 |
|  | 2012 | 4 | 25.0 | 8.9 | 51.6 – 13.9 | 1 |
| Folly Beach, SC | 2010 | 0 | - | - | - | - |
|  | 2011 | 15 | 18.8 | 0.9 | 23.5 – 12.9 | 0 |
|  | 2012 | 55 | 21.6 | 1.3 | 54.9 – 7.4 | 7 |

Table S2 continued

| Location | Year | n | Mean (mm) | SE | Range (mm) | # of individuals > 32.8 mm |
| --- | --- | --- | --- | --- | --- | --- |
| Murrells Inlet, SC | 2010 | 0 | - | - | - | - |
|  | 2011 | 4 | 15.1 | 4.0 | 26.3 -9.1 | 0 |
|  | 2012 | 4 | 15.4 | 3.0 | 21.5 – 9.4 | 0 |
| Wrightsville Beach, NC | 2010 | 0 | - | - | - | - |
|  | 2011 | 4 | 12.6 | 0.9 | 15.2 – 10.7 | 0 |
|  | 2012 | 10 | 18.1 | 2.2 | 28.8 – 10.1 | 0 |
| Frisco Beach, NC | 2010 | 0 | - | - | - | - |
|  | 2011 | 4 | 12.8 | 3.0 | 19.3 – 4.9 | 0 |
|  | 2012 | 53 | 20.0 | 1.3 | 41.7 – 6.7 | 8 |
| Avon, NC | 2010 | 0 | - | - | - | - |
|  | 2011 | 48 | 17.4 | 1.0 | 29.6 – 5.8 | 0 |
|  | 2012 | 30 | 13.4 | 1.0 | 36.2 – 4.8 | 1 |
| Rodanthe, NC | 2010 | 0 | - | - | - | - |
|  | 2011 | 14 | 16.2 | 1.5 | 28.9 – 5.5 | 0 |
|  | 2012 | 370 | 10.7 | 0.2 | 35.5 -1.5 | 1 |
| Kill Devil Hills, NC | 2010 | 0 | - | - | - | - |
|  | 2011 | 0 | - | - | - | - |
|  | 2012 | 0 | - | - | - | - |
